# Maternal Blood Pressure in Relation to Prenatal Lipid-Based Nutrient Supplementation and Adverse Birth Outcomes in a Ghanaian Cohort: A Randomized Controlled Trial and Cohort Analysis

**DOI:** 10.1093/jn/nxab018

**Published:** 2021-03-10

**Authors:** Alyssa M Abreu, Rebecca R Young, Ashley Buchanan, Ingrid E Lofgren, Harriet E T Okronipa, Anna Lartey, Per Ashorn, Seth Adu-Afarwuah, Kathryn G Dewey, Brietta M Oaks

**Affiliations:** Department of Nutrition and Food Sciences, University of Rhode Island, Kingston, RI, USA; Department of Nutrition, University of California, Davis, Davis, CA, USA; Department of Pharmacy Practice, College of Pharmacy, University of Rhode Island, Kingston, RI, USA; Department of Nutrition and Food Sciences, University of Rhode Island, Kingston, RI, USA; Department of Nutrition, University of California, Davis, Davis, CA, USA; Department of Nutrition and Food Science, University of Ghana, Legon, Ghana; Center for Child Health Research, Faculty of Medicine and Health Technology, Tampere University, Tampere, Finland; Department of Nutrition and Food Science, University of Ghana, Legon, Ghana; Department of Nutrition, University of California, Davis, Davis, CA, USA; Department of Nutrition and Food Sciences, University of Rhode Island, Kingston, RI, USA

**Keywords:** birth outcomes, Ghana, maternal blood pressure, prenatal supplements, maternal hypertension, low birth weight, preterm birth

## Abstract

**Background:**

It is unknown whether prenatal lipid-based nutrient supplements (LNSs) affect blood pressure (BP). Associations between hypertension and birth outcomes using recently updated BP cutoffs are undetermined.

**Objectives:**

We aimed to assess the impact of LNSs on maternal hypertension and associations between hypertension and birth outcomes.

**Methods:**

Pregnant Ghanaian women at ≤20 weeks of gestation (*n* = 1320) were randomly assigned to receive daily *1*) iron and folic acid (IFA), *2*) multiple micronutrients (MMN), or *3*) LNSs until delivery. BP was measured at enrollment and 36 weeks of gestation. We analyzed the effect of LNSs on BP using ANOVA and associations between hypertension [systolic BP (SBP) ≥130 mm Hg or diastolic BP (DBP) ≥80 mm Hg] and birth outcomes by linear and logistic regressions.

**Results:**

Mean ± SD SBP and DBP were 110 ± 11 and 63 ± 8 mm Hg at 36 weeks of gestation and did not differ by supplementation group (SBP, *P >* 0.05; DBP, *P >* 0.05). At enrollment, higher DBP was associated with lower birth weight and shorter gestation; women with high DBP had greater risk of low birth weight (LBW) [risk ratio (RR): 2.58; 95% CI: 1.09, 6.08] and preterm birth (PTB) (RR: 3.30; 95% CI: 1.47, 7.40). At 36 weeks of gestation, higher SBP was associated with lower birth weight, length, and head circumference and shorter gestation; higher DBP was associated with lower birth weight and length; and women with high DBP had greater risk of LBW (RR: 3.39; 95% CI: 1.32, 8.69). Neither high SBP nor hypertension were associated with birth outcomes at either time point.

**Conclusions:**

Daily provision of LNSs does not affect maternal hypertension, compared with IFA and MMN. Higher SBP and DBP are associated with a shorter gestation and smaller birth size; however, only high DBP is associated with LBW and PTB. The new BP cutoffs may help identify pregnancies at risk of adverse birth outcomes.

This trial was registered at clinicaltrials.gov as NCT00970866.

## Introduction

Hypertension is currently defined as a systolic blood pressure (SBP) ≥130 mm Hg or diastolic blood pressure (DBP) ≥80 mm Hg (1). The definition was recently updated by the American Heart Association, which previously designated an SBP ≥140 mm Hg or DBP ≥90 mm Hg as hypertension, based on the increased risk of cardiovascular disease in adult populations in the United States (1). Based on the previous hypertension cutoffs, >20% of pregnancies in Ghana are affected by hypertension (2), compared with ∼10% worldwide (3). The disparity may be related to lack of access to adequate health care, a delay in seeking health care, or delayed response to maternal health status at the health care facilities (4).

During a typical pregnancy, blood pressure (BP) decreases from early (<15 weeks of gestation) to mid-pregnancy (22–24 weeks of gestation), increases in late pregnancy (36 weeks of gestation), and then returns to prepregnancy levels by delivery (4, 5). However, during pregnancy BP may not change as expected, and hypertension may develop. Compared with antihypertensive medication, which may increase the risk of pregnancy complications (6, 7), nutritional supplements may be an effective and preferred strategy to decrease the risk of maternal hypertension.

Increased dietary intake of essential fatty acids (EFAs) has been shown to decrease the risk of hypertension in nonpregnant populations (8). Although the exact mechanisms between hypertension and dietary EFAs are unclear, EFAs have been associated with increased anti-inflammatory effects, antioxidation, endothelial vasodilation, vascular compliance, and inhibition of the renin–angiotensin–aldosterone system (8). There is emerging evidence that lipids, specifically EFAs, may play a role in decreasing placental dysfunction and inflammation, and supporting increased fetal growth (9, 10). However, EFAs have been associated with reduced risk of hypertension and inflammatory markers during pregnancy only in animal studies. Larger intervention studies have not shown significant associations between EFA supplementation or intake and maternal hypertension (11). There is also no evidence from African populations regarding the effects of nutrient supplements that include EFAs on maternal hypertension, although the prevalence of hypertensive disorders of pregnancy in Africa may be as high as 10%–25% (12).

It is unclear whether the new definition of hypertension, which uses a lower threshold, is appropriate for predicting pregnancies at risk of adverse outcomes. High BP during pregnancy, as defined by the previous cutoffs, has been associated with an increased risk of low birth weight (LBW) (13), small for gestational age (SGA), and preterm birth (PTB) (13, 14). It is important to minimize the risk of adverse birth outcomes because they have both short- and long-term consequences on newborn health. These consequences include infections (15), breathing difficulties (16), and increased risk of diabetes and obesity (17) and intellectual and developmental disabilities (18).

Ghana is a low-middle-income country located in Western Africa, with staple foods that include maize, cassava, rice, fish, and leafy vegetables. The country is currently experiencing a nutrition transition to more energy-dense, “Western” diets and corresponding increases in overweight and obesity. Several micronutrient deficiencies, including iron, folate, and vitamin A, are common in Ghana, and especially among lower-income households (19). Dietary intake of calcium was also reported to be low among women of childbearing age in the Manya Krobo District in the Eastern Region of Ghana (20). Calcium supplementation daily has been associated with a decreased risk of maternal hypertension, although evidence suggests that a protective effect is evident only among women with low dietary calcium intake (21). Given the high prevalence of hypertension in Ghana, it is important to identify effective strategies to decrease maternal BP and prevent associated adverse birth outcomes (22).

Our objectives were to *1*) evaluate the impact of prenatal lipid-based nutrient supplements (LNSs) on maternal BP; and *2*) assess the association between maternal BP during early and late pregnancy and birth outcomes using the new BP cutoffs. We hypothesized that women receiving LNSs would have a lower mean SBP and DBP than those receiving iron and folic acid (IFA) or multiple micronutrients (MMN) at 36 weeks of gestation. We also hypothesized that hypertension during pregnancy would be associated with a smaller newborn birth size and lower duration of gestation than in women with normal BP.

## Methods

### Study design

We report on a secondary data analysis of 1 of the randomized controlled trials in the International Lipid-Based Nutrient Supplements (iLiNS) Project, the iLiNS-DYAD trial in Ghana (23). A total of 1320 women were recruited from the Yilo and Manya Krobo districts of Ghana, randomly assigned to 1 of 3 groups, and received *1*) IFA supplements that contained 60 mg Fe and 400 μg folic acid, *2*) MMN that contained 1–2 RDAs of 18 vitamins and minerals, or *3*) small-quantity LNSs that contained the same micronutrients as the MMN as well as calcium, phosphorus, potassium, magnesium, EFAs, and other macronutrients from enrollment until delivery. **Table 1** provides the composition of the supplements, as well as a comparison to current RDAs during pregnancy (24–29). The main objective of this trial was to determine the effects of nutrient supplements for the mother during pregnancy and the first 6 mo postpartum, and for the child from 6–18 mo, on child growth through 18 mo of age. For this secondary analysis, data collected from enrollment, with a mean gestational age of 16 wk, through birth were used (23).

**TABLE 1:** Composition of supplements provided to pregnant women in the International Lipid-Based Nutrient Supplements-DYAD trial in Ghana^1^

Nutrient	RDA (pregnancy)	IFA	MMN	LNS
Ration, g/d			1 tablet	20
Total energy, kcal			0	118
Protein, g			0	2.6
Fat, g			0	10
Linoleic acid, g	13*		0	4.59
α-Linolenic acid, g	1.4*		0	0.59
Vitamin A (retinyl acetate), μg RE	770		800	800
Vitamin C (l-ascorbic acid), mg	85		100	100
Thiamin (thiamin hydrochloride), mg	1.4		2.8	2.8
Riboflavin, mg	1.4		2.8	2.8
Niacin (niacinamide), mg	18		36	36
Folic acid (pteroyl monoglutamic acid), μg	600	400	400	400
Pantothenic acid (calcium pantothenate), mg	6*		7	7
Vitamin B-6 (pyridoxine hydrochloride), mg	1.9		3.8	3.8
Vitamin B-12 (cyanocobalamin 0.1%), μg	2.6		5.2	5.2
Vitamin D (cholecalciferol), IU	600		400	400
Vitamin E (dl-α-tocopherol acetate), mg	15		20	20
Vitamin K (phylloquinone 5%), μg	90*		45	45
Iron (ferrous sulfate), mg	27	60	20	20
Zinc (zinc sulfate), mg	11		30	30
Copper (encapsulated copper sulfate), mg	1.0		4	4
Calcium (tricalcium phosphate), mg	1000		0	280
Phosphorus (tricalcium phosphate), mg	700		0	190
Potassium (potassium chloride), mg	2900*		0	200
Magnesium (magnesium citrate), mg	350		0	65
Selenium (sodium selenite 1.5%), μg	60		130	130
Iodine (potassium iodate), μg	220		250	250
Manganese (manganese sulfate), mg	2.0*		2.6	2.6

1 IFA capsule is standard of care and follows the WHO and Ghana Health Service recommendation; LNS for pregnant and lactating women (24–29); MMN supplement capsule (30). *Adequate intake. IFA, iron and folic acid; LNS, lipid-based nutrient supplement; MMN, multiple micronutrients; RE, retinol equivalents.

The small-quantity (20 g/d) LNS provided to women in this trial was a paste designed to be mixed with local foods to increase the nutrient and energy content of diets during pregnancy and lactation (30). Nutriset SAS produced the LNS in 20-g sachets and DSM South Africa produced the capsules of the IFA and MMN supplements. A more detailed description of the study population and methods has previously been published (23). The iLiNS-DYAD study protocol was approved by the institutional review boards at the University of California, Davis; the Noguchi Memorial Institute for Medical Research, University of Ghana; and the Ghana Health Service.

Eligibility for this study was specific to women attending 4 prenatal clinics in the Yilo and Manya Krobo districts of Ghana between December 2009 and December 2011, who were ≥18 y old, and at ≤20 weeks of gestation as determined by ultrasound. Reasons for exclusion were a test result that was HIV positive, residence > 20 km outside of the study area, history of peanut or milk allergies, severe illness, or the intention to move out of the study area within 2 y.

A block-randomization scheme was designed and implemented by a study statistician and has been detailed previously (23). Each woman was randomly assigned into the IFA, MMN, or LNS group. To ensure blinding, an independent party from the research team color-coded the supplement capsules for the IFA and MMN groups that were then provided to blinded investigators, fieldworkers, and participants. Because the LNS was not a capsule, fieldworkers and participants could not be blinded from distinguishing between the LNS and IFA or MMN. Anthropometrists were not aware of the group allocations, and data analysts were blinded until the completion of preliminary analyses.

### Procedures

At enrollment and 36 weeks of gestation, trained fieldworkers interviewed participants to record sociodemographic characteristics. A study nurse measured height (Seca 217), weight (Seca 874), and duplicate BP measurements on either arm using automated BP monitors (Andon BPM model KD-595). We collected a blood sample by venipuncture and measured hemoglobin (Hb; Hemocue AG), malaria parasitemia (Clearview Malarial Combo, Vision Biotech), and the inflammatory markers C-reactive protein (CRP; mg/L) and α1-acid glycoprotein (AGP; g/L) (Cobas Integra 400 plus Automatic Analyzer, Roche Diagnostic Corp.) from plasma. Gestational age estimates were determined at enrollment by physicians at the antenatal clinics using ultrasound imagers (EDAN DUS 3 Digital Ultrasonic Diagnostic Imaging System, EDAN Instruments, Inc.). Fieldworkers distributed a 2-wk supply of the assigned supplement along with instructions on consumption methods. Fieldworkers collected data on supplement adherence, calculated as the percentage of pregnancy days after enrollment that women reported consuming the supplement, and morbidity at biweekly follow-up visits at the participants’ homes.

Fieldworkers visited the home or hospital at delivery to complete anthropometric measurements of newborns. Birth weight was measured to the nearest 20 g (Seca 383; Seca), and length (Seca 416; Seca) and head circumference (Shorr Productions) to the nearest 0.1 cm. Measurements were recorded within 48 h of birth for 91% of infants and between 3 and 14 d after birth for 9% of infants. For age- and sex-standardization of weight, we used the WHO Child Growth Standards (31). If the infant was measured >48 h after delivery, adjustments for weight, length, and head circumference were calculated as previously described (23).

### Outcomes and definitions

The primary outcome for this secondary analysis of the effect of the intervention was mean maternal SBP at 36 weeks of gestation. We also examined mean DBP at 36 weeks of gestation, and risk of high SBP or high DBP at 36 weeks of gestation by supplement group. We defined hypertension as high SBP (≥130 mm Hg) or high DBP (≥80 mm Hg) (1). To examine the association between maternal BP and birth outcomes, our primary outcome was newborn birth weight. Additional outcomes included newborn birth length, head circumference, pregnancy duration, LBW (<2500 g), PTB (<37 wk), stunting (length-for-age *z* score <−2), and SGA (birth weight <10th percentile according to the Intergrowth Standards) (32). PTB was examined only with respect to measurements of BP taken at enrollment because many PTBs occurred before the BP measurements at 36 weeks of gestation.

### Statistical analysis

#### Effect of daily nutrient supplementation on maternal hypertension

For continuous outcomes, the difference between the 3 group means was tested with ANOVA and ANCOVA models. If the null hypothesis was rejected at the 0.05 significance level, post hoc pairwise comparisons across the 3 intervention groups were conducted using the Tukey–Kramer test for ANOVA to adjust for multiple comparisons. Log-binomial models were used to estimate risk ratios (RRs) (33). If a model did not converge, a log-Poisson model was used (33). Normality for all variables was assessed using a Shapiro–Wilk statistic. At enrollment, prepregnancy BMI, CRP, and AGP were not normally distributed and were logarithmically transformed for analysis. The heteroscedasticity assumption was examined through the plots and we observed relatively equal variances. No outliers were identified through visual inspection of the histograms or scatterplots. For each outcome, we evaluated possible covariates to control for confounding by 16 prespecified variables: maternal (age, height, BMI, completed years of education, marital status, parity, gestational age, season, treatment group, Hb status, CRP, AGP, and malaria status), child (sex), and household (socioeconomic status indicators—namely, assets and food insecurity). Covariates that were statistically significantly associated with the outcome (*P* < 0.1 in univariate models) were included in an adjusted regression model. Among all the variables included in the final adjusted model, there was no evidence of collinearity [variance inflation factors (VIFs) <10] and all variables significantly associated with the outcome were included in adjusted models. Owing to randomization of the trial in the intervention, we followed the intention-to-treat principle when analyzing the effect of LNSs on maternal BP. During the study, some of the IFA and MMN capsules were unintentionally mislabeled, causing 92 IFA women and 85 MMN women to have mixed exposure during pregnancy. All women pregnant during the period of mixed exposure were excluded from this analysis including the 92 IFA and 85 MMN women who experienced mixed exposure, as well as 86 LNS women. After exclusion the final sample size was 1057 (23). A sensitivity analysis including all participants (*n* = 1320) was also conducted. Considering the protocol violation resulting in mixed exposure, the analyses included intervention groups according to the supplement treatment assignment actually received when they were enrolled and not the treatment originally assigned at enrollment, which is consistent with a previous iLiNS-DYAD publication reporting birth size (23). All tests were 2-sided and conducted at a 0.05 level of significance.

#### Association between maternal hypertension and birth outcomes

A prospective cohort study design was used to analyze the associations between maternal BP (at enrollment and 36 weeks of gestation) and birth outcomes. Multiple linear regression models were used to determine the associations of SBP and DBP with birth weight, length, head circumference, and duration of gestation. We checked all models for linearity using quadratic terms and found a lack of any U-shaped relations. Results of the regression models are presented as standardized regression coefficients (β-coefficients), which allow for standardized comparisons between the predictors and the outcome variables. Using β-coefficients, a 1-SD increase in the predictor determines the change in SDs of the outcome variable, with all other variables held constant. Interactions between maternal BMI and BP, with respect to birth outcomes, were assessed in linear regression and *P* values < 0.05 were considered to be statistically significant. Log-binomial models were used to estimate RR, 95% CIs, and *P* values for categorical birth outcomes in adjusted and unadjusted models using both the old and new definitions of high SBP, high DBP, and hypertension (33). In a few instances, the models did not converge and log-Poisson models were used (33). The categorical birth outcomes included LBW, SGA, PTB, and stunting. If the null hypothesis was rejected at the 0.05 level, the Benjamini–Hochberg procedure was used to compare *P* values to an adjusted significance level that accounted for multiple tests related to birth outcomes (34). Inclusion of covariates was used and tested as aforementioned. Both CRP and AGP were included as potential covariates owing to the association between increased maternal inflammation and decreased newborn birth size (35).

SAS software 9.4 (SAS Institute, Cary, NC) was used for data analysis in addition to Microsoft Office Excel for the configuration of figures.

## Results

**FIGURE 1:**
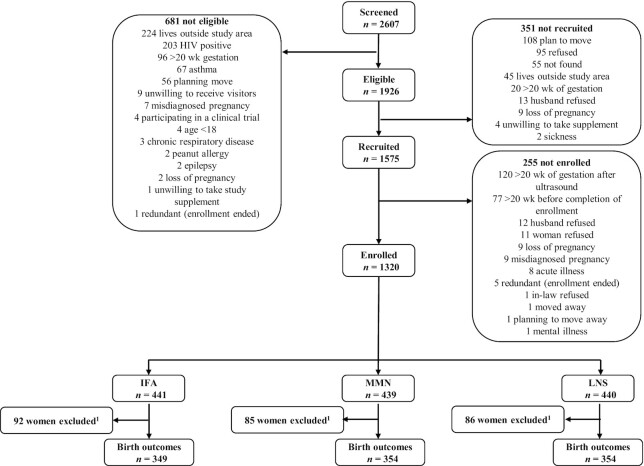
Study profile. The IFA group received 60 mg iron plus 400 mg folic acid. The MMN group received 1–2 Recommended Dietary Allowances of 18 vitamins and minerals (including 20 mg iron). The LNS group received LNS with the same micronutrients as the MMN group, plus another 4 minerals (Ca, P, K, and Mg), as well as macronutrients. All 3 supplements were intended for daily consumption. ^1^During the study, IFA and MMN capsules were unintentionally mislabeled, causing 92 participants in the IFA group and 85 participants in the MMN group to receive the incorrect supplement. A total of 86 women not-exposed in the LNS group, as well as the mixed-exposure women in the IFA or MMN groups were excluded.

Between December 2009 and March 2011, a total of 1320 pregnant women were enrolled in the main iLiNS-DYAD trial, with a final sample size of 1057 for this analysis after excluding women who were pregnant during the mixed-exposure period (****Figure 1****). Only 4.4% of women dropped out of the study before delivery and the attrition rate did not significantly differ (*P* > 0.05) between the IFA (4.6%), MMN (3.7%), and LNS (5.1%) groups (23). **Table 2** shows maternal characteristics at enrollment, by intervention group. At enrollment, 8.1% of women had hypertension (SBP ≥130 mm Hg or DBP ≥80 mm Hg) with 6.6% of women having high SBP and 3.6% having high DBP. At 36 weeks of gestation, 5.3% of women had hypertension with 4.3% having high SBP and 2.4% having high DBP. **Supplemental Table 1** presents the characteristics of the total sample of women, as well as those with normal BP and with hypertension at enrollment. Women with hypertension were older on average and had greater prepregnancy BMI and height than those without hypertension (*P* < 0.05).

**TABLE 2:** Characteristics of pregnant Ghanaian women enrolled between 2009 and 2011 in the International Lipid-Based Nutrient Supplements-DYAD nutrient supplementation trial, by supplement group^1^

Characteristic	IFA	MMN	LNS
*n*	349	354	354
Maternal age, y	26.5 ± 5	26.9 ± 6	26.5 ± 5
Gestational age, wk	16.3 ± 3	16.2 ± 3	16.2 ± 3
Parous, %	62	69	64
BMI, kg/m^2^	24.5 ± 4	24.4 ± 4	24.7 ± 4
Height, cm	158.5 ± 6	159.1 ± 6	159.0 ± 5
Education, completed years	7.6 ± 4	7.5 ± 4	7.7 ± 4
Married or cohabitating, %	92	94	93
Offspring sex female, %	49	51	49
Plasma CRP, mg/L	3.8 (3.3, 4.3)	3.1 (2.8, 3.5)	3.3 (2.9, 3.8)
Plasma AGP, g/L	0.6 (0.6, 0.7)	0.6 (0.6, 0.6)	0.6 (0.6, 0.6)
Positive malaria test, %	9	8	11
SBP, mm Hg	112 ± 10	111 ± 12	112 ± 11
DBP, mm Hg	63 ± 7	64 ± 8	64 ± 8
High SBP, %	5	8	7
High DBP, %	3	3	5
HTN, %	7	9	8

1 *n* = 1057. Values presented are mean ± SD or geometric mean (95% CI) unless otherwise indicated. IFA capsule is standard practice and follows the WHO and Ghana Health Service recommendation; LNS for pregnant and lactating women (24–29); MMN supplement capsule (30). AGP, α1-acid glycoprotein; CRP, C-reactive protein; DBP, diastolic blood pressure; HTN, hypertension; IFA, iron and folic acid; LNS, lipid-based nutrient supplement; MMN, multiple micronutrients; SBP, systolic blood pressure.

### Supplement group comparisons

**Table 3** shows that the unadjusted and adjusted means of SBP and DBP at 36 weeks of gestation were not significantly different between intervention groups. There were no statistically significant differences in risk of maternal hypertension between the intervention groups (**Table 4**). In the sensitivity analysis including all 1320 pregnant women, neither SBP nor DBP differed significantly by supplementation group in unadjusted or adjusted analysis (data not shown).

**TABLE 3:** Unadjusted mean SBP and DBP at 36 weeks of gestation in pregnant Ghanaian women enrolled between 2009 and 2011 in the International Lipid-Based Nutrient Supplements-DYAD randomized trial of daily nutrient supplementation in a semiurban setting, by intervention group^1^

	IFA	MMN	LNS	*P* value
*n*	349	354	354	
SBP, mm Hg				
Unadjusted	110 ± 10	110 ± 11	110 ± 11	0.704
Adjusted^2^	110 ± 10	110 ± 11	110 ± 11	0.958
DBP, mm Hg				
Unadjusted	63 ± 7	62 ± 8	63 ± 8	0.266
Adjusted^2^	63 ± 7	62 ± 8	63 ± 8	0.668

1 *n* = 1057. Values are mean ± SD. IFA capsule is standard practice and follows the WHO and Ghana Health Service recommendation; LNS for pregnant and lactating women (24–29); MMN supplement capsule (30). DBP, diastolic blood pressure; IFA, iron and folic acid; LNS, lipid-based nutrient supplement; MMN, multiple micronutrients; SBP, systolic blood pressure.

2 The adjusted models presented the same means as the unadjusted models. Confounding variables that had a statistically significant association with the outcome (*P* < 0.1) were included in an adjusted regression model. Adjusted models included prepregnancy BMI, gestational age, maternal age, completed years of education, asset index, food insecurity index, hemoglobin status, maternal height, plasma C-reactive protein, plasma α1-acid glycoprotein, parity, and malaria status; all covariates were ascertained at study enrollment.

**TABLE 4:** Risk of high SBP or DBP at 36 weeks of gestation in pregnant Ghanaian women enrolled between 2009 and 2011 in the International Lipid-Based Nutrient Supplements-DYAD randomized trial of daily nutrient supplementation in a semiurban setting, between intervention groups^1^

	IFA	MMN	LNS	RR^1^ (95% CI)	RR (95% CI)
*n*	349	354	354		
High SBP, *n* (%)	12 (3.4)	14 (4.0)	19 (5.4)		
Unadjusted				1.56 (0.77, 3.17)	1.36 (0.69, 2.66)
Adjusted^2^				1.37 (0.64, 2.92)	1.21 (0.61, 2.41)
High DBP, *n* (%)	6 (1.7)	8 (2.3)	11 (3.1)		
Unadjusted				1.81 (0.68, 4.83)	1.38 (0.56, 3.38)
Adjusted^2^				1.91 (0.89, 4.08)	1.10 (0.39, 3.06)

1 *n* = 1057. RR of high SBP (≥130 mm Hg) compared with normal SBP (<130 mm Hg), and high DBP (≥80 mm Hg) compared with normal DBP (<80 mm Hg). IFA capsule is standard practice and follows the WHO and Ghana Health Service recommendation; LNS for pregnant and lactating women (24–29); MMN supplement capsule (30). DBP, diastolic blood pressure; IFA, iron and folic acid; LNS, lipid-based nutrient supplement; MMN, multiple micronutrients; RR, risk ratio; SBP, systolic blood pressure.

2 Covariates that were significantly associated with the outcome (*P* < 0.1) were included in an adjusted regression model. Adjusted models included prepregnancy BMI, gestational age, maternal age, completed years of education, asset index, food insecurity index, hemoglobin status, maternal height, plasma C-reactive protein, plasma α1-acid glycoprotein, parity, and malaria status (for MMN comparison); all covariates were ascertained at study enrollment.

### Maternal BP and birth outcomes

#### BP as a continuous variable and birth outcomes

Higher DBP at enrollment was associated with lower birth weight (β: −0.087; 95% CI: −0.15, −0.020; *P* < 0.05) and shorter pregnancy duration (β: −0.069; 95% CI: −0.14, −0.0001; *P* = 0.05) in adjusted models (**Supplemental Table 2**). For each 1-SD increase in DBP at enrollment (8 mm Hg), birth weight was reduced by 0.087 SD, which translates to 37 g reduced birth weight per 1-SD increase in DBP. For each 1-SD increase in DBP at enrollment, duration of gestation was reduced by 0.069 SD, which translates to an ∼1-d reduction in gestation duration per 1-SD increase in DBP. When the model for birth weight was further adjusted for duration of gestation, the effect size was attenuated and no longer statistically significant (β: −0.057; 95% CI: −0.117, 0.003; *P >* 0.05).

Higher DBP at 36 weeks of gestation was associated with a lower birth weight (β: −0.095; 95% CI: −0.16, −0.027; *P* < 0.05) and length (β: −0.076; 95% CI: −0.14, −0.009; *P* < 0.05) in adjusted models. For each 1-SD increase in DBP at 36 weeks of gestation (8 mm Hg), birth weight was reduced by 0.095 SD, which translates to 41 g reduced birth weight per 1-SD increase in DBP. When the model for birth weight was further adjusted for duration of gestation, the effect size remained significant (β: −0.067; 95% CI: −0.13, −0.006; *P* < 0.05). Higher SBP at 36 weeks of gestation was associated with a lower birth weight (β: −0.074; 95% CI: −0.14, −0.008; *P* < 0.05), birth length (β: −0.077; 95% CI: −0.14, −0.011; *P* < 0.05), newborn head circumference (β: −0.072; 95% CI: −0.14, −0.002; *P* < 0.05), and a shorter pregnancy duration (β: −0.069; 95% CI: −0.14, −0.001; *P* < 0.05). When the model for birth weight was further adjusted for duration of gestation, the effect size was attenuated and no longer significant (β: −0.043; 95% CI: −0.10, −0.017; *P* > 0.05).

#### High BP, using the new cutoffs, and birth outcomes

Women with high DBP at enrollment had greater risk of LBW (adjusted RR: 2.58; 95% CI: 1.09, 6.08) and PTB (RR: 3.30; 95% CI: 1.47, 7.40) than women with normal DBP at enrollment (**Table 5**). Neither high SBP nor hypertension at enrollment were significantly associated with LBW in unadjusted or adjusted models (Table 5). Women with high DBP at 36 weeks of gestation had greater risk of LBW (RR: 3.39; 95% CI: 1.32, 8.69); however, neither high SBP nor hypertension at 36 weeks of gestation were associated with any birth outcomes.

**TABLE 5:** Risk of adverse birth outcomes predicted by maternal BP at enrollment and 36 weeks of gestation in pregnant Ghanaian women enrolled between 2009 and 2011 in the International Lipid-Based Nutrient Supplements-DYAD nutrient supplementation trial^1^

	Low birth weight^2^		Small for gestational age^3^		PTB^4^		Stunting^5^	
	RR (95% CI)	*P*	RR (95% CI)	*P*	RR (95% CI)	*P*	RR (95% CI)	*P*
*n* (%)	93 of 931 (10)		189 of 897 (21)		76 of 931 (8)		81 of 925 (9)	
Enrollment								
Normal SBP, *n*	88 of 987		180 of 987		70 of 987		78 of 987	
High SBP, *n*	5 of 70		9 of 70		6 of 70		3 of 70	
Unadjusted	0.81 (0.34, 1.92)	0.633	0.71 (0.38, 1.31)	0.276	1.22 (0.55, 2.70)	0.619	0.54 (0.18, 1.68)	0.289
Adjusted	1.02 (0.38, 2.76)	0.969	0.90 (0.47, 1.72)	0.761	1.29 (0.55, 3.03)	0.566	0.50 (0.13, 1.97)	0.321
Normal DBP, *n*	88 of 1019		180 of 1019		70 of 1019		80 of 1019	
High DBP, *n*	5 of 38		9 of 38		6 of 38		1 of 38	
Unadjusted	1.55 (0.67, 3.55)	0.304	1.31 (0.74, 2.32)	0.356	2.33 (1.09, 4.98)	0.029*	0.34 (0.05, 2.35)	0.273
Adjusted	2.58 (1.09, 6.08)	0.031*	1.72 (0.95, 3.10)	0.074	3.30 (1.47, 7.40)	0.004*	0.51 (0.07, 3.71)	0.503
Normal BP, *n*	86 of 971		176 of 971		67 of 971		78 of 971	
HTN, *n*	7 of 86		13 of 86		9 of 86		3 of 86	
Unadjusted	0.97 (0.47, 2.02)	0.937	0.87 (0.53, 1.45)	0.599	1.60 (0.83, 3.08)	0.157	0.46 (0.15, 1.41)	0.172
Adjusted	1.27 (0.56, 2.90)	0.563	1.01 (0.59, 1.75)	0.958	1.89 (0.92, 3.86)	0.083	0.40 (0.10, 1.61)	0.198
36 weeks of gestation								
Normal SBP, *n*	89 of 1012		180 of 1012		—		79 of 1012	
High SBP, *n*	4 of 45		9 of 45		—		2 of 45	
Unadjusted	1.03 (0.40, 2.66)	0.955	1.13 (0.63, 2.03)	0.682	—	—	0.58 (0.15, 2.25)	0.428
Adjusted	2.01 (0.77, 5.26)	0.156	1.67 (0.91, 3.06)	0.099	—	—	0.97 (0.25, 3.78)	0.966
Normal DBP, *n*	89/1032		184/1032				80/1032	
High DBP, *n*	4 of 25		5 of 25		—		1 of 25	
Unadjusted	2.05 (0.83, 5.03)	0.118	1.19 (0.55, 2.57)	0.655	—	—	0.57 (0.08, 3.86)	0.561
Adjusted	3.39 (1.32, 8.69)	0.011*	1.54 (0.74, 3.20)	0.250	—	—	0.86 (0.12, 6.24)	0.880
Normal BP, *n*	88 of 1001		179 of 1001		—		79 of 1001	
HTN, *n*	5 of 56		10 of 56		—		2 of 56	
Unadjusted	1.09 (0.47, 2.56)	0.838	1.06 (0.60, 1.86)	0.845	—	—	0.48 (0.12, 1.91)	0.300
Adjusted	2.07 (0.85, 5.04)	0.108	1.45 (0.81, 2.62)	0.212	—	—	0.77 (0.19, 3.05)	0.705

1 RR of high SBP (≥130 mm Hg) compared with normal SBP (<130 mm Hg), high DBP (≥80 mm Hg) compared with normal DBP (<80 mm Hg), and HTN (high SBP or high DBP) compared with normal BP. All covariates were ascertained at study enrollment. *Adjusted *P* value is statistically significant with Benjamini–Hochberg correction, *P* < 0.05. Critical values are as follows: unadjusted PTB at enrollment, 0.116; adjusted PTB at enrollment, 0.016; adjusted LBW at enrollment, 0.062; adjusted LBW at 36 weeks of gestation, 0.033. BP, blood pressure; DBP, diastolic blood pressure; HTN, hypertension; LBW, low birth weight; PTB, preterm birth; RR, risk ratio; SBP, systolic blood pressure; SGA, small for gestational age.

2 Adjusted SBP and DBP models for LBW included prepregnancy BMI, maternal age, asset index, food insecurity index, parity, offspring sex, and maternal height.

3 Adjusted SBP models for SGA included prepregnancy BMI, maternal age, completed school years, parity, maternal height, log plasma C-reactive protein, and malaria status. Adjusted DBP models for SGA included the same variables as SBP as well as log plasma α1-acid glycoprotein.

4 PTB is defined as delivery before 37 weeks of gestation. PTB was examined only with respect to measurements of BP taken at enrollment because many PTBs occurred before the BP measurements at 36 weeks of gestation. Adjusted SBP models for PTB included prepregnancy BMI, gestational age at enrollment, asset index, food insecurity index, season at enrollment being dry season, malaria status, and treatment group. Adjusted DBP models for PTB included prepregnancy BMI, gestational age at enrollment, asset index, food insecurity index, and season at enrollment being dry season.

5 Adjusted SBP and DBP models for stunting included prepregnancy BMI, maternal age, asset index, food insecurity index, parity, season at enrollment being dry season, and maternal height.

We observed variation in coefficients between unadjusted and adjusted models and examined which covariates had the greatest impact on results. BMI had a large influence in adjusted models. At enrollment, higher BMI was associated with higher SBP (β: 0.389; 95% CI: 0.330, 0.446; *P* < 0.05) and DBP (β: 0.375; 95% CI: 0.320, 0.432; *P* < 0.05). Higher BMI at enrollment was also associated with higher SBP (β: 0.401; 95% CI: 0.350, 0.457; *P* < 0.05) and DBP (β: 0.394; 95% CI: 0.340, 0.450; *P* < 0.05) at 36 weeks of gestation, and newborn birth weight (β: 0.266; 95% CI: 0.200, 0.328; *P* < 0.05). We also checked all models for collinearity and confirmed all VIFs were <2.

#### High BP, using previous cutoffs, and birth outcomes

When using the prior BP cutoff, women with high DBP (≥90 mm Hg) at enrollment had greater risk of LBW (RR: 8.50; 95% CI: 3.52, 20.57) and PTB (RR: 10.8; 95% CI: 4.87, 20.89) than women with normal DBP at enrollment. High SBP (≥140 mm Hg) at enrollment was not significantly associated with LBW in unadjusted or adjusted models (**Supplemental Table 3**). Women with high DBP at 36 weeks of gestation had greater risk of LBW (RR: 8.50; 95% CI: 3.52, 20.57); however, neither high SBP nor hypertension were associated with any birth outcomes.

## Discussion

In this study, we determined that provision of LNS did not have a significant effect on maternal BP during pregnancy, as compared with provision of IFA or MMN. We also examined associations between maternal BP and birth outcomes and found that higher DBP and higher SBP were both associated with lower birth weight and length and shorter pregnancy duration. However, only high DBP (≥80 mm Hg) was associated with increased risk of LBW and PTB, whereas high SBP, according to the new cutoff of ≥130 mm Hg, was not associated with risk of any adverse birth outcomes examined in this study.

Our findings that LNSs did not have a significant effect on maternal BP are consistent with a previous trial conducted in Bangladesh, which compared pregnant women consuming either LNSs or IFA and found no significant difference in mean SBP, DBP, or risk of hypertension (36). As was the case in our population, the Bangladesh study sample also had a low prevalence of hypertension as compared with the previously reported country prevalence. However, both studies excluded populations with chronic conditions, which may have led to a lower prevalence of hypertension. Women in this Ghanaian study population have been shown to have low urinary iodine (37), but adequate plasma fatty acid levels (38) and low prevalence of iron deficiency anemia (39). It is possible that LNSs would show an effect on maternal BP in populations with a higher prevalence of micronutrient deficiencies. The only other study to date that has examined the effects of LNSs on BP was a follow-up study of the children from our trial in Ghana which reported no effect of prenatal and early childhood LNSs on child BP at 4–6 y of age (40).

The associations we found in our study between BP and birth outcomes are also consistent with previous research. A 2014 systematic review/meta-analysis of 55 studies confirmed an association between hypertension and risk of LBW and PTB (14). Our findings show that when using the previous definition the association between hypertension at 36 weeks of gestation and LBW (RR: 3.8) is similar in magnitude to the estimate from the 2014 systematic review/meta-analysis (RR: 2.7) that included 7 different definitions of hypertension during pregnancy.

Maternal hypertension may lead to reduced placental perfusion, placental dysfunction, and/or increased inflammation (9, 41), which could explain the association we observed between hypertension and LBW. Inflammation may lead to fetal hypoxia, which may inhibit fetal growth and thereby reduce newborn birth weight (42). Alternatively, maternal hypertension may actually be a consequence of fetal growth restriction, because fetal growth restriction and placental dysfunction may decrease placental vasodilators, which are increasingly important during late-pregnancy BP maintenance (43). The exact underlying mechanisms for how maternal BP and birth weight are related remain unclear.

Interestingly, although both SBP and DBP were associated with lower birth weight, the magnitude of the association was noticeably greater for DBP. A study by Bakker et al. (13) also reported a greater magnitude of association for DBP and birth weight than for SBP in a large cohort study of pregnant women in the Netherlands. The functional differences between DBP and SBP may explain our finding. The heart muscles relax and the chambers fill with blood during diastole and contract to pump blood into the arteries during systole. Vascular restructuring occurs during pregnancy to increase blood flow and accommodate the needs of the fetus for growth and development. Less filling of the heart with blood will occur with higher DBP, which may result in decreased cardiac output and blood flow to the placenta for fetal growth and development (44).

The associations between DBP (at enrollment and 36 weeks of gestation) and birth weight were attenuated with an adjustment for duration of gestation, which suggests that duration of gestation may be a mediating factor in the relation between DBP and birth weight. However, the association between DBP at 36 weeks of gestation and birth weight was significant even after adjusting for duration of gestation.

Our findings show a positive relation between maternal BMI and birth weight, and also a positive relation between maternal BMI and BP. These associations may explain the substantial impact that maternal BMI has on adjusted results. This suggests that there are opposing pathways with regard to how maternal BMI might influence birth size. Higher BMI may lead to greater newborn birth size, but if the mother also develops high BP, that may counter the effect of higher BMI, because high BP is associated with a smaller birth size.

Strengths of our study include the large prospective design with BP measurements in early and late pregnancy, a low loss to follow-up, the use of ultrasound scans to determine gestational age, and the use of the most recent cutoff definitions for hypertension. A limitation of our study was that adherence to supplement consumption was based primarily on participant self-report. However, fieldworkers also visited the houses biweekly and counted any unconsumed supplements to assist in confirming adherence. In addition, participants were able to distinguish between small-quantity LNS sachets and IFA or MMN capsules. However, anthropometrists and data analysts were blinded to group assignments. It should be noted that maternal BP was not the main outcome of the iLiNS trial and it is possible that the quality of the BP measurements could have been improved. Either the left or right arm was used to take BP measurements, and it is possible that the duplicate measurements were not appropriately timed, i.e., they may have been taken within 5 min of each other. We did not have information related to the use of antihypertensive medications during pregnancy; however, it is likely that only a limited number of women, if any, were using such medications during pregnancy given the low prevalence of hypertension in this study. Lastly, the low prevalence of hypertension may introduce bias and may be due to the exclusion of women with chronic conditions, as previously mentioned. Future studies should further explore the etiology of maternal hypertension and associations with birth outcomes using the updated BP cutoffs.

Within a sample of Ghanaian women, this analysis examined the impact of prenatal supplementation on maternal hypertension risk using the new, more conservative hypertension threshold. We did not observe differential effects of daily prenatal LNS, MMN, or IFA supplementation on maternal hypertension risk. Thus, although LNS is shown to benefit maternal and newborn health in this population (37–39), our results do not support its use to prevent maternal hypertension. Independently of the supplement intervention, however, we found that higher BP was associated with lower birth weight, length, and head circumference and shorter pregnancy duration, and maternal hypertension was associated with an increased risk of LBW and PTB. This suggests that the updated diagnostic threshold for hypertension may be a useful criterion for identifying women at greater risk of adverse birth outcomes, and that efforts to address hypertension during pregnancy to improve birth outcomes are needed.

## Supplementary Material

nxab018_Supplemental_File

## Notes

Supported by Bill & Melinda Gates Foundation grant OPP49817 to the University of California, Davis (to KGD).

Author disclosures: the authors report no conflicts of interest.

Supplemental Tables 1–3 are available from the “Supplementary data” link in the online posting of the article and from the same link in the online table of contents at https://academic.oup.com/jn/.

Abbreviations used: AGP, α1-acid glycoprotein; BP, blood pressure; CRP, C-reactive protein; DBP, diastolic blood pressure; EFA, essential fatty acid; Hb, hemoglobin; IFA, iron and folic acid; iLiNS, International Lipid-Based Nutrient Supplements; LBW, low birth weight; LNS, prenatal lipid-based nutrient supplement; MMN, multiple micronutrients; PTB, preterm birth; RR, risk ratio; SBP, systolic blood pressure; SGA, small for gestational age; VIF, variance inflation factor.
