# Supplementary material for: Maternal Blood Pressure in Relation to Prenatal Lipid-Based Nutrient Supplementation and Adverse Birth Outcomes in a Ghanaian Cohort: A Randomized Controlled Trial and Cohort Analysis
Source: J Nutr. 2021 Mar 10;151(6):1637–45. doi: 10.1093/jn/nxab018 (PMC8169808; doi:10.1093/jn/nxab018)
Supplement: nxab018_Supplemental_File [file nxab018_supplemental_file.docx]

**Title: Maternal blood pressure in relation to prenatal lipid-based nutrient supplementation and adverse birth outcomes in a Ghanaian cohort.**

**First Author: Alyssa M. Abreu**

**Online Supplementary Material**

**Supplemental Table 1:** Characteristics of pregnant Ghanaian women (n=1057) enrolled between 2009-2011 in the iLiNS-DYAD nutrient supplementation trial, by total, normal, and high blood pressure groups.^1^

| Characteristic | **Total** | **Normal BP Group** | **HTN Group** | ***p*** |
| --- | --- | --- | --- | --- |
| *n* | 1057 | 1001 | 56 |  |
| Maternal Age, years | 26.7 (5) | 26.6 (5) | 28.6 (6) | 0.007 |
| Gestational Age, wk | 16.2 (3) | 16.2 (3) | 16.3 (3) | 0.857 |
| Parity |  |  |  | 0.190 |
| Parous, % | 65 | 65 | 73 |  |
| BMI, kg/m^2^ | 24.5 (4) | 24.3 (4) | 28.6 (6) | <0.001 |
| Height, cm | 158.9 (6) | 158.8 (6) | 160.5 (6) | 0.023 |
| Education, completed years | 7.6 (3) | 7.6 (4) | 7.7 (4) | 0.833 |
| Married or cohabitating, % | 93% | 93% | 93% | 0.989 |
| Offspring Sex |  |  |  | 0.496 |
| Female, % | 50 | 50 | 45 |  |
| CRP, mg/L | 3.4 [3.2-3.7] | 3.4 [3.1-3.6] | 4.8 [3.4-6.6] | 0.092 |
| AGP, g/L | 0.6 [0.6-0.6] | 0.6 [0.6-0.6] | 0.7 [0.6-0.7] | 0.089 |
| SBP, mmHg | 112 (11) | 110 (10) | 135 (9) | <0.001 |
| DBP, mmHg | 64 (8) | 63 (7) | 80 (8) | <0.001 |
| Positive Malaria test, % | 10 | 10 | 10 | 0.782 |

^1^Values presented are mean (SD) or geometric mean [95% CI] unless otherwise indicated. BMI, body mass index; CRP, plasma C-reactive protein; AGP, plasma α1-acid glycoprotein; SBP, systolic blood pressure; DBP, diastolic blood pressure; BP, blood pressure; HTN, hypertension- SBP ≥ 130 mmHg or DBP ≥ 80 mmHg

**Supplemental Table 2:** Adjusted standardized regression coefficients (𝛃) of systolic and diastolic blood pressure predictors at enrollment and 36 weeks gestation and birth outcomes in pregnant Ghanaian women (n=1057) enrolled between 2009-2011 in the iLiNS-DYAD nutrient supplementation trial.^1^

| One standard deviation | Birth Weight^2^  0.43 kg  Adjusted 𝛃 (95% CI) | Birth Length^3^  1.97 cm  Adjusted 𝛃 (95% CI) | Head Circ.^4^  1.38 cm  Adjusted 𝛃 (95% CI) | Gestational Age^5^  1.94 wk  Adjusted 𝛃 (95% CI) |
| --- | --- | --- | --- | --- |
| *n* | 930 | 925 | 925 | 997 |
| Blood Pressure at Enrollment  SBP, mmHg | -0.062 (-0.128, 0.004) | -0.062 (-0.128, 0.003) | -0.058 (-0.127, 0.010) | -0.051 (-0.118, 0.016) |
| *p* | 0.066 | 0.064 | 0.097 | 0.136 |
| DBP, mmHg | -0.087 (-0.154,-0.020) | -0.064 (-0.130, 0.003) | -0.037 (-0.106, 0.032) | -0.069 (-0.138, 0.000) |
| *p* | 0.011* | 0.062 | 0.290 | 0.050* |
| Blood Pressure at 36 wk  SBP, mmHg | -0.074 (-0.141,-0.008) | -0.077 (-0.143,-0.011) | -0.072 (-0.141,-0.002) | -0.069 (-0.136,-0.001) |
| *p* | 0.029* | 0.023* | 0.043* | 0.048* |
| DBP, mmHg | -0.095 (-0.162,-0.027) | -0.076 (-0.144,-0.009) | -0.057 (-0.126, 0.012) | -0.061 (-0.131, 0.008) |
| *p* | 0.006* | 0.026† | 0.106 | 0.084 |

^1^Abbreviations: SBP, systolic blood pressure; DBP, diastolic blood pressure; BP, blood pressure. *P,* *P*-value at 0.05 significance level. If the null-hypothesis was rejected at the 0.05 level, the Benjamini-Hochberg procedure was used to compare *P*-values to an adjusted significance level that accounts for multiple tests related to birth outcomes. * *P-*value is significant with Benjamini-Hochberg correction. † *P-*value is not significant with Benjamini-Hochberg correction.

^2^Adjusted SBP and DBP models for birth weight included pre-pregnancy BMI, maternal age, asset index, parity, hemoglobin status, offspring sex, maternal height, and treatment group; all covariates were ascertained at study enrollment.

^3^Adjusted SBP and DBP models for birth length included the same as for birth weight and season at enrollment being dry season; all covariates were ascertained at study enrollment.

^4^Adjusted SBP and DBP models for head circumference included pre-pregnancy BMI, maternal age, parity, offspring sex, maternal height, and treatment group; all covariates were ascertained at study enrollment.

^5^Adjusted SBP and DBP models for gestational age included pre-pregnancy BMI, gestational age, maternal age, asset index, food insecurity index, hemoglobin status, and offspring sex; all covariates were ascertained at study enrollment. **Supplemental Table 3.** Risk of adverse birth outcomes^1^ predicted by maternal blood pressure (BP) using Pre-2017 BP cutoffs (SBP ≥140 mmHg or DBP ≥90 mmHg) at enrollment and 36 weeks gestation in pregnant Ghanaian women enrolled between 2009-2011 in the iLiNS-DYAD nutrient supplementation trial.^1^

|  |  | Low Birth Weight^2^ | |  | Small for Gestational Age^3^ | |  | Preterm^4^ | |
| --- | --- | --- | --- | --- | --- | --- | --- | --- | --- |
|  |  | RR^2^ (95%CI) | *p* |  | RR (95%CI) | *p* |  | RR (95%CI) | *p* |
| ***n* (%)** |  | 93/931 (10) |  |  | 189/897 (21) |  |  | 76/931 (8) |  |
| **Enrollment** |  |  |  |  |  |  |  |  |  |
| Normal SBP, *n* |  | 92/1044 |  |  | 187/1044 |  |  | 75/1044 |  |
| High SBP, *n* |  | 1/13 |  |  | 2/13 |  |  | 1/13 |  |
| Unadjusted |  | 0.83 (0.13, 5.49) | 0.849 |  | 0.79 (0.22, 2.81) | 0.715 |  | 1.02 (0.15, 6.75) | 0.983 |
| Adjusted |  | 1.68 (0.27, 10.35) | 0.575 |  | 1.41 (0.44, 4.57) | 0.565 |  | 1.72 (0.31, 9.41) | 0.533 |
|  |  |  |  |  |  |  |  |  |  |
| Normal DBP, *n* |  | 91/1052 |  |  | 188/1052 |  |  | 74/1052 |  |
| High DBP, *n* |  | 2/5 |  |  | 1/5 |  |  | 2/5 |  |
| Unadjusted |  | 5.09 (1.88, 13.83) | 0.001* |  | 1.19 (0.22, 6.51) | 0.843 |  | 6.26 (2.29, 10.89) | <0.001* |
| Adjusted |  | 8.50 (3.52, 20.57) | <0.001* |  | 1.82 (0.48, 6.85) | 0.376 |  | 10.08 (4.87, 20.89) | <0.001* |
|  |  |  |  |  |  |  |  |  |  |
| Normal BP, *n* |  | 91/1042 |  |  | 187/1042 |  |  | 74/1042 |  |
| HTN, *n* |  | 2/15 |  |  | 2/15 |  |  | 2/15 |  |
| Unadjusted |  | 1.55 (0.43, 5.64) | 0.504 |  | 0.73 (0.20, 2.62) | 0.626 |  | 1.91 (0.52, 6.96) | 0.327 |
| Adjusted |  | 3.22 (0.37, 11.44) | 0.071 |  | 1.26 (0.38, 4.19) | 0.703 |  | 3.26 (0.95, 11.24) | 0.061 |
|  |  |  |  |  |  |  |  |  |  |
| **36 weeks** |  |  |  |  |  |  |  |  |  |
| Normal SBP, *n* |  | 92/1047 |  |  | 188/1047 |  |  |  |  |
| High SBP, *n* |  | 1/10 |  |  | 1/10 |  |  |  |  |
| Unadjusted |  | 1.00 (0.15, 6.49) | 0.999 |  | 0.47 (0.07, 3.04) | 0.430 |  | -- | -- |
| Adjusted |  | 1.99 (0.33-11.92) | 0.454 |  | 0.89 (0.16, 5.06) | 0.894 |  | -- | -- |
|  |  |  |  |  |  |  |  |  |  |
| Normal DBP, *n* |  | 91/1053 |  |  | 188/1053 |  |  |  |  |
| High DBP, *n* |  | 2/4 |  |  | 1/4 |  |  |  |  |
| Unadjusted |  | 5.09 (1.88, 13.83) | 0.001* |  | 1.19 (0.22, 6.51) | 0.843 |  | -- | -- |
| Adjusted |  | 8.50 (3.52, 20.57) | <0.001* |  | 1.82 (0.48, 6.85) | 0.376 |  | -- | -- |
|  |  |  |  |  |  |  |  |  |  |
| Normal BP, *n* |  | 91/1046 |  |  | 188/1046 |  |  |  |  |
| HTN, *n* |  | 2/11 |  |  | 1/11 |  |  |  |  |
| Unadjusted |  | 1.84 (0.52, 6.54) | 0.347 |  | 0.43 (0.07, 2.79) | 0.375 |  | -- | -- |
| Adjusted |  | 3.76 (1.08, 13.11) | 0.038† |  | 0.78 (0.13, 4.54) | 0.779 |  | -- | -- |

^1^Risk ratio of High SBP (≥ 140 mmHg) compared to Normal SBP (< 140 mmHg), High DBP (≥ 90 mmHg) compared to Normal DBP (< 90mmHg), and HTN (High SBP or High DBP) compared to Normal BP. SBP, systolic blood pressure; DBP, diastolic blood pressure; BP, blood pressure; HTN, High SBP or High DBP; *P,* *P*-value at 0.05 significance level. If the null-hypothesis was rejected at the 0.05 level, the Benjamini-Hochberg procedure was used to compare *P*-values to an adjusted significance level that accounts for multiple tests related to birth outcomes. *Adjusted *P-*value is significant after Benjamini-Hochberg procedure. †Adjusted *P-*value is not significant after Benjamini-Hochberg procedure. All covariates were ascertained at study enrollment. Stunting results are not available because there were no stunted newborns among women with high SBP or high DBP at enrollment or 36 weeks.

^2^ Adjusted SBP and DBP models for LBW included pre-pregnancy BMI, maternal age, asset index, food insecurity index, parity, offspring sex, and maternal height.

^3^ SBP models for SGA included pre-pregnancy BMI, maternal age, completed school years, parity, maternal height, log C-reactive protein from plasma, and malaria status. DBP models for SGA included the same variables as SBP as well as log AGP.

^4^Preterm is defined as delivery prior to 37 wk gestation. Preterm birth was examined only with respect to measurements of BP taken at enrollment because many preterm births occurred before the 36 wk BP measurements. SBP models for preterm birth included pre-pregnancy BMI, gestational age at enrollment, asset index, food insecurity index, season at enrollment being dry season, malaria status, and treatment group. DBP models for preterm birth included pre-pregnancy BMI, gestational age at enrollment, asset index, food insecurity index, season at enrollment being dry season.
